# A real-world study on the safety profile of extended-interval dosing of immune checkpoint inhibitors for melanoma: a single-center analysis in Japan

**DOI:** 10.3389/fmed.2023.1293397

**Published:** 2023-12-07

**Authors:** Takamichi Ito, Yumiko Kaku-Ito, Fumitaka Ohno, Takeshi Nakahara

**Affiliations:** Department of Dermatology, Graduate School of Medical Sciences, Kyushu University, Fukuoka, Japan

**Keywords:** acral melanoma, Asian population, nivolumab, PD-1, pembrolizumab

## Abstract

**Background:**

Anti-programmed death-1 (PD-1) antibodies are the mainstay for the treatment of unresectable or high-risk melanoma. However, real-world data on the safety profile of their extended-interval doses (EDs) are limited, particularly in Asian patients with melanoma.

**Materials and methods:**

In this single-center retrospective study, we analyzed the risks of immune-related adverse events (irAEs) among 71 Japanese patients (36 males; mean age, 65.0 years) who received anti-PD-1 monotherapy for melanoma at our institute. Patients who were administered ipilimumab prior to anti-PD-1 monotherapy were excluded. Patients were divided into three groups: canonical-interval dose (CD) group (*n* = 50, body weight-based dosing or 240 mg Q2W for nivolumab and body weight-based dosing or 200 mg Q3W for pembrolizumab), ED group (*n* = 14, 480 mg Q4W for nivolumab and 400 mg Q6W for pembrolizumab), and dose-switch (DS) group (*n* = 7, upfront CD followed by ED).

**Results:**

The CD group received nivolumab more frequently in the metastatic setting. There were no significant differences in baseline characteristics among the three groups, including in sex, age, primary tumor site, tumor subtype, and follow-up period. irAEs occurred in 36.6% (26 patients) of all patients (32.0% of the CD group, 35.7% of the ED group, and 71.4% of the DS group), while severe (grade ≥ 3) irAEs occurred in only two patients, both of whom were in the CD group. Most of the irAEs occurred during the first 6 months of anti-PD-1 therapy and, interestingly, all of the irAEs in the DS group occurred before the switch (during the CD). There was no significant difference among the three groups in the probability of irAE estimated by the Kaplan–Meier method.

**Conclusion:**

These findings may highlight the safety of ED of anti-PD-1 monotherapy in the treatment of Asian patients with melanoma.

## 1 Introduction

Since the first introduction of immune checkpoint inhibitors (ICIs) for malignant melanoma, they have revolutionized the management of melanoma and led to dramatic improvements in patient survival (1–3). The application of ICIs has rapidly expanded to other cancers, hematologic malignancies, and sarcomas. Programmed death-1 (PD-1) is a key molecule of immune checkpoints and its inhibitors are now the mainstay of melanoma treatment in both metastatic and adjuvant settings (1–7). Clinical practice guidelines recommend anti-PD-1 therapy alone or with other drugs (e.g., ipilimumab) as first-line treatment for unresectable melanoma and high-risk advanced melanoma, particularly for BRAF wild-type melanoma (1–3). Melanoma subtypes differ between Caucasian and Asian populations, with Caucasians having more sunlight-related melanomas and Asians having more acral and nail melanomas (8–10). There are different genetic backgrounds for these subtypes, which can lead to differences in their biological behavior and response to antitumor therapy (11–15). There is evidence suggesting that acral melanoma is refractory to ICIs, and even non-acral cutaneous melanoma has a worse prognosis in Asians than in Caucasians under ICI therapy (16). These results indicate that response to ICIs varies depending on the tumor subtype and ethnicity.

Two anti-PD-1 inhibitors, nivolumab and pembrolizumab, have been approved for use in treating melanoma in Japan (2, 17). Nivolumab was initially used for every 2 weeks (Q2W) at a body weight-based dosing or a flat dosage of 240 mg, and later for its extended-interval dose (ED) of 480 mg every 4 weeks (Q4W). Pembrolizumab, on the other hand, was initially used for every 3 weeks (Q3W) at a body weight-based dosing or a flat dosage of 200 mg, and subsequently approved for its ED [400 mg every 6 weeks (Q6W)]. The approval of these drugs was based on pharmacokinetic data obtained from prior studies (18–21). While ED with anti-PD-1 antibodies would be convenient by reducing clinical visits, administering ED may be associated with increased risks for immune-related adverse events (irAEs). To date, only limited real-world evidence of the safety of ED has been obtained, particularly for Asian patients with melanoma (22–26). Is ED of anti-PD-1 monotherapy safe for Asians with melanoma? Is it necessary to initiate anti-PD-1 monotherapy with the canonical-interval dose (CD) and later switch to ED to reduce irAEs? This single-center retrospective study was conducted to answer these questions. Interestingly, no clear increase in irAEs or severe (grade ≥ 3) irAEs was observed in our cohort treated with ED compared with CD.

## 2 Methods

### 2.1 Ethics statement

We conducted this retrospective study in accordance with the concepts enshrined in the Declaration of Helsinki. This study was approved by Kyushu University Institutional Ethics Committee (30-363; 27 November, 2018). Written informed consent was received from the patients prior to their inclusion in the study.

### 2.2 Patients

The study included a total of 71 patients with malignant melanoma who received anti-PD-1 monotherapy (nivolumab and/or pembrolizumab) in a metastatic or adjuvant setting at the Department of Dermatology, Kyushu University (Fukuoka, Japan), between July 2014 and March 2023. Patients who received anti-CTLA4 therapy (monotherapy or in combination with anti-PD1 antibody) prior to anti-PD-1 monotherapy were excluded. Patients who received other anti-tumor treatments, including BRAF/MEK inhibitors, cytotoxic chemotherapy, and interferon β, prior to anti-PD-1 monotherapy were included. No patients underwent simultaneous anti-PD-1 plus any of these anti-tumor therapies including BRAF/MEK inhibitor therapy. At least three experienced dermatopathologists confirmed the diagnosis of all patients.

The following clinical and demographic data on all patients were retrieved from the patients’ clinical records and analyzed: age at the initiation of anti-PD-1 monotherapy, sex, primary tumor site, tumor subtype, type of anti-PD-1 antibody, lines of treatment, types of irAEs and their grades (CTCAE v.5.0), and timing of irAEs. Two authors (T.I. and Y.K.-I.) independently reviewed the records of all patients included in this study and any discrepancy in the results that they recorded was resolved through discussion.

Patients were divided into three groups, namely, CD group, ED group, and dose-switch (DS) group. The CD group included patients who received the original doses of nivolumab (2 mg/kg Q3W, 3 mg/kg Q2W, and 240 mg Q2W) and pembrolizumab (2 mg/kg Q3W and 200 mg Q3W) throughout the course of anti-PD-1 monotherapy. The ED group included patients receiving ED (480 mg Q4W for nivolumab and 400 mg Q6W for pembrolizumab) from the beginning to the end of anti-PD-1 monotherapy. The DS group consisted of patients who started with CD and later switched to ED.

### 2.3 Statistical analysis

All statistical analyses were performed using GraphPad Prism version 8.3 (GraphPad Software, San Diego, CA, United States). To analyze the relationship among the three groups, chi-squared test and Kruskal–Wallis test were used for categorical and continuous variables, respectively. The Kaplan–Meier method and the log-rank test were used to estimate the probability of irAE. Patients who did not experience any irAE were censored at the last follow-up. A *p*-value of less than 0.05 was considered to indicate statistical significance.

## 3 Results

### 3.1 Patient clinicopathological data

Baseline characteristics of all 71 patients are shown in Table 1. All patients were Japanese (36 males and 35 females), with a mean age of 65.0 years (median, 69; range 30–86). Primary tumors were located on the skin of the extremities (43.7%), followed by non-skin lesions such as mucosa or viscera (25.4%), trunk skin (14.1%), head and neck skin (8.5%), and those of unknown origin (8.5%). Non-acral cutaneous melanoma was the predominant subtype (36.6%), followed by acral melanoma (28.2%), mucosal melanoma (14.1%), and uveal melanoma (7.0%). Melanoma of unknown origin or unclassified type was found in 14.1%. Nivolumab monotherapy or pembrolizumab monotherapy was performed in 47.9 and 49.3% of the patients, respectively. Two patients received both nivolumab and pembrolizumab monotherapy in a sequential setting. Approximately 65% of patients received the therapy in a metastatic setting (49.3% as 1st line, 11.3% as 2nd line, and 2.8% as 3rd line or more) and 36.6% of patients in an adjuvant setting. The mean follow-up periods after the initiation of anti-PD-1 therapy were 91.8 weeks (median, 68 weeks; range 4–443 weeks) for all patients, 91.5 weeks (median, 52 weeks; range 4–443 weeks) for the CD group, 73.4 weeks (median, 91 weeks; range 6–134 weeks) for the ED group, and 131.0 weeks (median, 130 weeks; range 14–259 weeks) for the DS group. There was no significant difference in the follow-up period among the three groups (*p* = 0.224).

**Table 1:** Baseline characteristics.

	All patients (*n* = 71)	Canonical-interval dose (*n* = 50)	Extended-interval dose (*n* = 14)	Dose switch^a^ (*n* = 7)	*p* value
Sex, *n* (%)					0.369
Male	36 (50.7)	28 (56.0)	5 (35.7)	3 (42.9)	
Female	35 (49.3)	22 (44.0)	9 (64.3)	4 (57.1)	
Age, y					0.599
Mean (SD)	65.0 (14.0)	64.0 (15.0)	65.2 (12.5)	71.3 (5.8)	
Median (Min, Max)	69 (30, 86)	67 (30, 88)	67 (43, 83)	72 (63, 80)	
Primary site, *n* (%)					0.467
Head and neck	6 (8.5)	5 (10.0)	1 (7.1)	0 (0)	
Trunk	10 (14.1)	5 (10.0)	3 (21.4)	2 (28.6)	
Extremities	31 (43.7)	23 (46.0)	6 (42.9)	2 (28.6)	
Non-skin	18 (25.4)	14 (28.0)	3 (21.4)	1 (14.3)	
Unknown	6 (8.5)	3 (6.0)	1 (7.1)	2 (28.6)	
Tumor subtype, *n* (%)					0.844
Non-acral cutaneous	26 (36.6)	19 (38.0)	5 (35.7)	2 (28.6)	
Acral	20 (28.2)	14 (28.0)	4 (28.6)	2 (28.6)	
Mucosal	10 (14.1)	6 (12.0)	3 (21.4)	1 (14.3)	
Uveal	5 (7.0)	5 (10.0)	0 (0)	0 (0)	
Others/unknown	10 (14.1)	6 (12.0)	2 (14.3)	2 (28.6)	
Tumor stage, *n* (%)					<0.001
II	4 (5.6)	0 (0)	3 (21.4)	1 (14.3)	
III	15 (21.1)	6 (12.0)	6 (42.9)	3 (42.9)	
IV	52 (73.2)	44 (88.0)	5 (35.7)	3 (42.9)	
Treatment, *n* (%)					0.004^c^
Nivolumab	34 (47.9)	30 (60.0)	3 (21.4)	1 (14.3)	
Pembrolizumab	35 (49.3)	18 (36.0)	11 (78.6)	6 (85.7)	
Sequential^b^	2 (2.8)	2 (4.0)	0 (0)	0 (0)	
Line of treatment, *n* (%)					<0.001^d^
1st line^e^	36 (50.7)	32 (64.0)	2 (14.3)	2 (28.6)	
2nd line^e^	8 (11.3)	6 (12.0)	1 (7.1)	1 (14.3)	
3rd line or more^e^	2 (2.8)	2 (4.0)	0 (0)	0 (0)	
Adjuvant	25 (35.2)	10 (20.0)	11 (78.6)	4 (57.1)	
Treatment cycles					0.033
Mean (SD)	10.2 (10.0)	9.5 (8.2)	6.8 (4.7)	22.0 (19.2)	
Median (Min, Max)	8 (1, 62)	7 (1, 42)	7 (2, 16)	15 (3, 62)	
Follow-up period, w					0.224
Mean (SD)	91.8 (91.3)	91.5 (101.7)	73.4 (44.4)	131.0 (77.5)	
Median (Min, Max)	68 (4, 443)	52 (4, 443)	91 (6, 134)	130 (14, 259)	

a Switch from canonical interval dose to extended interval dose.

b Sequential use of nivolumab and pembrolizumab.

c Compared between nivolumab and pembrolizumab.

d Compared between metastatic and adjuvant setting.

e Metastatic setting.

Comparing the three groups (CD, ED, and DS), there were no significant differences in sex, age, primary tumor site, or tumor subtype (Table 1). Nivolumab was more frequently used in the CD group and pembrolizumab was more frequently used in the ED group and the DS group. In addition, a metastatic setting was more common in the CD group and an adjuvant setting was more common in the ED group and the DS group. There were significant differences in the AJCC tumor stage (8th edition) and treatment cycles among the three groups.

### 3.2 Adverse events

Comprehensive profiles of irAEs are summarized in Table 2. In total, 26 events of any grade occurred in the follow-up period, namely, 11 endocrinopathy-related events (thyroid dysfunction, adrenal dysfunction, and diabetes), along with 7 cutaneous, 2 pneumonitis, 2 fatigue, 1 hepatitis, 1 musculoskeletal, 1 ocular, and 1 gastrointestinal irAEs. Cutaneous irAE included 4 maculopapular rash, 1 psoriasiform dermatitis, 1 vitiligo, and 1 edema. Notably, only two severe (grade 3) irAEs (type 1 diabetes and hepatitis) occurred in all patients. Anti-PD-1 monotherapy was discontinued in two patients due to an irAE (grade 3 type 1 diabetes in one patient and grade 2 edema in the other), and the most common reason for terminating anti-PD-1 therapy was disease progression. The patient who experienced grade 3 hepatitis resumed the anti-PD-1 therapy after a temporary interruption. No patient switched back to CD.

**Table 2:** Adverse events.

	All patients (*n* = 71)		Canonical-interval dose (*n* = 50)		Extended-interval dose (*n* = 14)		Dose switch^a^ (*n* = 7)			
	Total events		Total events		Total events		Total events		Escalation window^b^	
Adverse event	Any grade	Grade ≥ 3	Any grade	Grade ≥ 3	Any grade	Grade ≥ 3	Any grade	Grade ≥ 3	Any grade	Grade ≥ 3
All events, *n* (%)	26 (36.6)	2 (2.8)	16 (32.0)	2 (4.0)	5 (35.7)	0	5 (71.4)	0	0	0
Endocrinopathy	11	1^c^	5	1^c^	3		3			
Skin	7		5		1		1			
Pneumonitis	2		1				1			
Fatigue	2		2							
Hepatitis	1	1^d^	1	1^d^						
Musculoskeletal	1		1							
Ocular	1		1							
Gastrointestinal	1				1					

a Switch from canonical interval dose to extended interval dose.

b Period after switch from canonical interval dose to extended interval dose.

c Type 1 diabetes, Grade 3.

d Grade 3.

In the CD group, a total of 16 irAEs including the two severe irAEs occurred, while no severe irAEs occurred in the ED group or the DS group. Interestingly, all five irAEs in the DS group occurred before the switch and no irAEs were identified during the subsequent ED period (escalation window).

### 3.3 Probability of irAE

**Figure 1:**
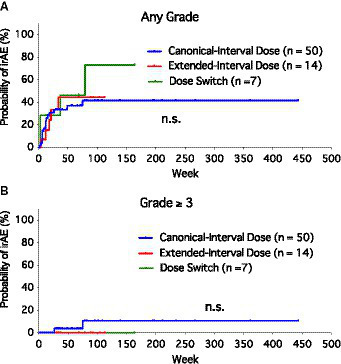
Kaplan–Meier curves of the probability of immune-related adverse events (irAEs) among canonical-interval dose group, extended-interval dose group, and dose-switch group. **(A)** Any grade of irAEs. **(B)** Severe (grade ≥ 3) irAEs.

Since most patients experienced only one irAE event or a second irAE at nearly the same timing as the first, we created Kaplan–Meier curves to compare the risk of irAEs among the groups (Figure 1). There was no significant difference in the probability of irAE (free of irAEs of any grade) among the three groups (Figure 1A). Considering the severe irAEs (grade ≥ 3), no significant difference was found among the three groups as well (Figure 1B). Most of the irAEs occurred during the first 6 months after the initiation of anti-PD-1 monotherapy (mean, 18.8 weeks; median, 12 weeks; range, 2–79 weeks).

## 4 Discussion

In this retrospective study, we found that ED had safety comparable to that of CD. Overall, 32.0% of patients treated with CD experienced an irAE of any grade, while the corresponding value was 35.7% for the ED group. Severe irAEs of grade 3 or more occurred exclusively in the CD group. Most of the irAEs occurred during the first 6 months after the initiation of anti-PD-1 therapy. All of the irAEs in the DS group occurred before the switch to ED.

The use of ICIs has significantly impacted the clinical practice of medical oncology. Despite their first introduction as traditional body weight-based dosing regimens, simulation pharmacokinetics studies showed that weight has only a minor effect on the distribution of ICIs; therefore, flat ICI doses became standard (27–29). Data from clinical trials also indicate that ICIs with ED (pembrolizumab 400 mg Q6W and nivolumab 480 mg Q4W) offer similar outcomes and safety as CD schedules (200 mg Q3W and 240 mg Q2W, respectively) (18, 20, 21).

However, there have been scarce real-world data on the safety profile of ED, particularly for Asian patients with melanoma (22–26). A retrospective study in Japan examined 45 patients with non-small cell lung cancer treated with pembrolizumab. All patients started at the CD and switched to ED after a median of six cycles of CD. New irAEs or the deterioration of existing ones occurred in 37.8% within three cycles of ED after switching, and the authors concluded that the ED may induce new irAEs (particularly pneumonitis) during the first few cycles after the switch, even in patients who had received stable treatment at CD (22). Another study from Japan retrospectively investigated the safety of ED of nivolumab and pembrolizumab across 69 patients with various solid cancers (including 21 melanomas) (23). Among 60 patients who switched to ED, 13 patients (21.7%) developed irAEs after the switch, seven of whom (53.8%) did so during the first ED cycle. These two studies may highlight the potential safety risk of ED.

In contrast, some recent reports have suggested that ED has a comparable safety profile to CD (24–26). A single-center analysis in the Netherlands compared the safety and efficacy between CD (*n* = 88) and ED cohorts (*n* = 117) with non-small cell lung cancer. Toxicity leading to dose reduction or discontinuation of treatment was not increased in the ED cohort (treatment was permanently discontinued due to irAEs in 4.3% of those on ICI treatment with ED) (24). Another study multicentrically recruited patients (*n* = 91) to analyze the safety of ED of ICIs for non-small cell lung cancer (25). After a median follow-up of 10.7 months on ICIs, only 4.3% of patients discontinued the treatment permanently, while 16% interrupted the treatment due to irAEs. More recently, a large cohort study on 812 patients with solid cancer (including 456 melanomas) was reported (26). Patients had received at least one cycle of monotherapy with ED after switching from CD or were treated upfront with ED. Out of 550 patients who started ICIs with CD and switched to ED, 225 (41%) developed irAEs of any grade and 17 (3%) those of grade 3 or more during CD, whereas irAEs of any grade and grade 3 or more were experienced by 155 (36%) and 20 (5%) patients after switching to ED, respectively. A lower probability of any grade irAEs was associated with switching to ED (adjusted odds ratio, 0.83; 95% confidence interval, 0.64–0.99; *p* = 0.047), whereas no significant difference was noted for ≥grade 3 events (adjusted odds ratio, 1.55; 95% confidence interval, 0.81 to 2.94; *p* = 0.18). The authors concluded that switching ICI treatment from CD and ED did not increase the incidence of irAEs. Our data, suggesting the unnecessity of dosing switch, align well with these studies (22–26). However, the reason behind the conflicting results (22–26) regarding the safety of extended dosing is unclear. One potential explanation could be the different irAE profiles among the cancers, such as frequent pneumonitis in lung cancer and vitiligo in melanoma.

ED may have several potential disadvantages such as less monitoring for clinical progression, negative impact on detecting irAEs. Increased economic cost is another potential disadvantage because the treatment will be stopped upon disease progression regardless of when the last dose was received, potentially leading to drug waste in the bloodstream, more likely in the ED group than the CD group (30). Occasional case reports of severe irAEs after dose switch have been published (31). Careful monitoring can help overcome these potential disadvantages and highlight clear benefits of ED.

Besides the potential biases inherent in the retrospective nature of this study and its small sample size especially in the ED and DS groups, a limitation of this study was the inability to analyze the efficacy profile of ED due to the significant involvement of adjuvant therapy. In addition, caution should be taken when interpreting our results due to the frequent use of ED in the adjuvant setting.

In conclusion, we have provided further insights into the safety profile of ED in the treatment of melanoma. Based on our data, the risk of irAEs was not increased with ED compared with CD. Dose switch with upfront CD followed by ED may not be necessary to reduce irAEs. With careful monitoring, especially during the early phase of anti-PD-1 monotherapy, the use of ED should be a safe and convenient strategy for treating melanoma in both adjuvant and metastatic settings.

## Data availability statement

The raw data supporting the conclusions of this article will be made available by the authors, without undue reservation.

## Ethics statement

The studies involving humans were approved by Kyushu University Ethics Committee. The studies were conducted in accordance with the local legislation and institutional requirements. The participants provided their written informed consent to participate in this study.

## Author contributions

TI: Conceptualization, Data curation, Formal analysis, Funding acquisition, Resources, Writing – original draft. YK-I: Data curation, Methodology, Writing – review & editing. FO: Methodology, Resources, Writing – review & editing. TN: Supervision, Validation, Writing – review & editing.
